# Dynamic change of PaO_2_ may affect the association between hyperoxemia and prognosis

**DOI:** 10.1186/s13054-023-04721-1

**Published:** 2023-11-09

**Authors:** Yanfei Shen

**Affiliations:** Department of Intensive Care, Zhejiang Hospital, 1229 Gudun Road, Hangzhou, 310013 Zhejiang China

## Introduction

I am writing to provide a comment on the recent article [1] published in Critical Care, which investigated the dose–response association between hyperoxemia and 30-day survival in cardiac arrest patients. A total of 9735 patients were included and categorized into mild (13.4–20 kPa), moderate (20.1–30 kPa), severe (30.1–40 kPa), and extreme (> 40 kPa), and normoxemia as PaO2 8–13.3 kPa. The reported findings, suggesting a potential association between hyperoxemia after reperfusion and improved prognosis in cardiac arrest patients, mark a significant departure from the established literature [2–4], which reported opposite findings that hyperoxemia was associated with worse prognosis in patients after cardiac arrest. Several factors may cause these inconsistent findings.

First, according to the current international guidelines [5], 100% inspired oxygen was often supplied during cardiopulmonary resuscitation (CPR) and after the return of spontaneous circulation until the arterial oxygen saturation can be measured reliably. Therefore, in real clinical situations, patients were often supplied with high-concentration oxygen from the beginning of resuscitation until the patients were admitted to the intensive care unit (ICU). These clinical procedures may cause hyperoxemia, but only in patients with mild cardiopulmonary dysfunction. In patients with severe cardiopulmonary dysfunction (e.g., massive lung exudation, cardiogenic shock, etc.), even high-concentration oxygen therapy is difficult to lead to hyperoxemia. Thus, to a certain degree, transient hyperoxemia in these patients may represent relatively good cardiopulmonary function after cardiac arrest. Therefore, there is the possibility that the association between transient hyperoxemia and lower mortality may be a reflection of the association between cardiopulmonary function and mortality.

Secondly, understanding the complexities of oxygen status assessment is pivotal in guiding clinical decision-making and ensuring optimal patient care. In clinical practice, oxygen partial pressure can be easily affected by parameters in mechanical ventilation [6], such as inspired oxygen fraction, minute ventilation volume, etc. Thus, relying solely on a static value of partial pressure of oxygen (PaO_2_) fails to capture the intricate dynamics of oxygenation in critically ill patients, and this limitation is particularly significant in the context of cardiac arrest patients, who often exhibit dynamic and rapidly changing physiological states. One recent study [7] analyzed the longitudinal PaO_2_ data of 2,028 patients with brain injury. Based on the dynamic value of PaO_2_ within 72 h, they identified three PaO_2_ trajectories: Traj-1 (mild hyperoxia), Traj-2 (transient severe hyperoxia), and Traj-3 (persistent severe hyperoxia). The multivariable model revealed that the risk of death was higher only in patients with Traj-3 (persistent severe hyperoxia), but was similar for patients with Traj-1 (mild hyperoxia) and Traj-2 (transient severe hyperoxia). Although the study cohorts are different, the study also suggests that transient hyperoxia may have different effects on outcomes than sustained hyperoxia. This finding underscores the dynamic nature of oxygen status and the inherent variability in its trajectories, which may significantly influence patient outcomes. Moreover, these variations in PaO_2_ trajectories emphasize the need for a comprehensive approach to oxygen management that extends beyond a singular focus on static PaO_2_ values.

Third, the complex interplay between oxygen delivery, consumption, and utilization in these patients necessitates a comprehensive approach to oxygenation assessment. Factors such as tissue perfusion, oxygen extraction, and the potential for oxygen toxicity should be carefully considered to obtain a more nuanced understanding of the true oxygen status and its impact on patient outcomes. For instance, continuous monitoring of tissue oxygenation, as proposed by Rose et al. [8] and Allen et al. [9], enables real-time adjustments to oxygen therapy based on individual patient parameters, thereby preventing the potential consequences of both hypoxia and hyperoxia [10].

In light of these considerations, I encourage further research that incorporates a multidimensional assessment of oxygen status, including markers of tissue perfusion and oxygen utilization, to elucidate the complex relationship between hyperoxemia and prognosis in cardiac arrest patients. A comprehensive understanding of these dynamics is crucial for the development of evidence-based guidelines that can effectively guide clinical practice and improve patient outcomes. Finally, this study contributed significantly to oxygen management in patients after cardiac arrest, and this work is very much appreciated!

## Data Availability

Not applicable.
